# COVID-19 vaccine uptake and associated factors among pregnant women attending antenatal care in Debre Tabor public health institutions: A cross-sectional study

**DOI:** 10.3389/fpubh.2022.919494

**Published:** 2022-07-19

**Authors:** Endeshaw Chekol Abebe, Gebrehiwot Ayalew Tiruneh, Getachew Asmare Adela, Teklie Mengie Ayele, Zelalem Tilahun Muche, Awgichew Behaile T/Mariam, Anemut Tilahun Mulu, Edgeit Abebe Zewde, Nega Dagnaw Baye, Tadesse Asmamaw Dejenie

**Affiliations:** ^1^Department of Biomedical Sciences, College of Health Sciences, Debre Tabor University, Debre Tabor, Ethiopia; ^2^Department of Midwifery, College of Health Sciences, Debre Tabor University, Debre Tabor, Ethiopia; ^3^Department of Reproductive Health and Nutrition, School of Public Health, Woliata Sodo University, Woliata Sodo, Ethiopia; ^4^Department of Pharmacy, College of Health Sciences, Debre Tabor University, Debre Tabor, Ethiopia; ^5^Department of Medical Biochemistry, College of Medicine and Health Sciences, University of Gondar, Gondar, Ethiopia

**Keywords:** pregnant women, COVID-19 vaccine uptake, associated factors, health institutions, Northwest Ethiopia

## Abstract

**Background:**

Vaccination is the best means of reducing the increased risk of severe COVID-19 during pregnancy. Data on COVID-19 vaccine uptake among pregnant women in Ethiopia is scarce. Thus, this study aimed to assess COVID-19 vaccine uptake and associated factors among pregnant women.

**Method:**

An institution-based cross-sectional study was conducted among 634 pregnant women attending antenatal care in Debre Tabor public health institutions from March 14 to 30, 2022. Participants were recruited using a multistage sampling technique and data were collected *via* face-to-face interviews using a pre-tested structured questionnaire. Stata version 16.0 software was used for data analysis. Multiple logistic regression analysis was used to assess factors associated with COVID-19 vaccine uptake, with a p-value< 0.05 considered statistically significant.

**Result:**

Only 14.4% (95% CI: 11.7%-17.3%) of participants had received at least one dose of COVID-19 vaccines. The main reasons for declining vaccination were fear that the COVID-19 vaccine may have harmful side effects on the fetus or the mother. Being 45 or older (AOR: 1.75, 95%CI: 1.01–2.95), being married (AOR: 1.26, 95%CI: 1.12, 2.96), having good knowledge (AOR:3.52, 95%CI:1.83–3.87), and a positive attitude (AOR:4.81, 95% CI: 1.42–7.33) were positive predictors of COVID-19 vaccine uptake. But attaining a college or university education (AOR: 0.43, 95%CI: 0.12–0.69) was found to be a barrier to vaccine uptake by pregnant women.

**Conclusion:**

COVID-19 vaccination among pregnant women was substantially low. Old age, being married, low education, good knowledge, and a positive attitude were significant predictors of COVID-19 vaccine uptake. To enhance the COVID-19 vaccine uptake, the government should improve the knowledge and attitude of pregnant women toward the COVID-19 vaccine.

## Introduction

COVID-19 vaccination is one of the most successful and cost-effective public health intervention strategies to mitigate the spread of SARS-CoV-2 and reduce the emergence of new strains (1–4). Reports have shown that vaccination significantly reduces global morbidity and mortality related to COVID-19 (5, 6). COVID-19 vaccination is currently considered an important public health priority to end the pandemic by developing effective immunity against COVID-19 both at the individual and community levels (7). Community protection by vaccination against COVID-19 spread, known as herd immunity, is achieved when at least 75% of the population gets vaccinated, highlighting the need for vaccination on a large scale (8).

Pregnant women are especially vulnerable during the current COVID-19 pandemic because they are at an increased risk of morbidity and mortality from COVID-19 (9, 10). Pregnant women are generally at a greater risk of severe illness, hospitalization, admission to the intensive care unit (ICU), invasive mechanical ventilation, preeclampsia, and death when compared to non-pregnant women with COVID-19 (1, 11–15). Compared to pregnant women without COVID 19, pregnant women with COVID-19 have also a higher risk of adverse birth outcomes such as preterm birth, stillbirth, cesarean delivery, and neonatal ICU admissions, implying a high likelihood of neonatal morbidity and mortality (11, 13, 14, 16–18). Vertical transmission has also been observed in a few cases in SARS-CoV-2 positive pregnant women, albeit it is extremely rare (19). Hence, vaccination against COVID-19 is found to be the best way to protect pregnant women (and the fetus) from serious illness or consequences (14).

Despite the fact that pregnant women were not involved in the initial clinical trials of the COVID-19 vaccine, current solid evidence on vaccine effectiveness and safety suggests that receiving a COVID-19 vaccine far outweighs any possible risk of vaccination during pregnancy (20). Another large body of data from studies done in countries where large numbers of pregnant women were vaccinated also indicates that COVID-19 vaccination during pregnancy is safe for both the mother and fetus, with very rare side effects and pregnancy-specific safety concerns (21–23). Recent clinical data on the safety of the COVID-19 vaccine during pregnancy also observed no difference in side effects between pregnant and non-pregnant women after vaccination (20, 24, 25).

A growing body of evidence shows that COVID-19 vaccination during pregnancy is found to be highly effective, equivalent to nonpregnant people, in preventing severe illness, hospitalization, and death from COVID-19 (26). The vaccine protects against the risk of developing severe COVID-19 in pregnant women by conferring strong protective immunity (14). Vaccination also builds immunity that offers protection for the fetus or neonate against COVID-19 *via* passive transplacental transfer of antibodies from the immunized mother to the fetus during pregnancy or to the newborn during lactation (15, 27). In light of the beneficial role of the vaccine for the mother, fetus, and baby with few or no adverse effects, major guidelines indicate that pregnant women are eligible for and can get any of the WHO approved COVID-19 vaccines and recommend COVID-19 vaccination during pregnancy (14, 15, 20, 28). Consequently, many countries around the world, including Ethiopia, nowadays strongly advise COVID-19 vaccination for people who are pregnant, trying to get pregnant now, or might become pregnant in the future to protect them from COVID-19 (20, 29–31).

COVID-19 vaccination program in Ethiopia commenced on 13 March 2021 by providing priority to health professionals and the elderly. On 16 November 2021, the Ethiopian Federal Ministry of Health (MoH) started a COVID-19 vaccination campaign aimed at vaccinating all people aged 12 years and above, including pregnant women, to end the pandemic (32). AstraZeneca, Janssen, Pfizer-BioNTech, and Sinopharm are currently available vacci nes in Ethiopia for the campaign.

Despite the recommendations of COVID-19 vaccination, low vaccine acceptance is becoming a growing global challenge, hindering vaccine uptake. Low vaccine acceptance among pregnant women is evident from studies in different countries, such as Saudi Arabia (50%) (33), Jordan (37%) (34), the US (41%) (35), Turkey (37%) (36), and other large-scale studies involving 16 countries (52.0%) (37). This is due to a global rise in COVID-19 vaccine hesitancy that lowers COVID-19 vaccine acceptance and uptake, especially during pregnancy. In practice, the COVID-19 vaccination rate among pregnant women was found to be low in different countries, such as Saudi Arabia (57.1%) (10), Scotland (32.3%) (31), the US (40%) (29), and England (53.7%) (38). Similarly, a prior study done in Ethiopia conducted among pregnant women to assess their willingness to receive COVID-19 vaccine (if the vaccines were available) indicated that a significant proportion of pregnant women were not willing to receive the vaccine for various reasons if the vaccination started (5). However, data on the actual practice of pregnant women in receiving the COVID-19 vaccine in Ethiopia is not available. Hence, this study aimed to assess COVID-19 vaccine uptake and associated factors among pregnant women. The findings from this study could help women, clinicians, and policymakers to make decisions and increase COVID-19 vaccine uptake during pregnancy.

## Methods and materials

### Study design, period, and setting

An institution-based cross-sectional study was conducted from March 14 to 30, 2022 at Debre Tabor public health institutions in Debre Tabor, Northwest Ethiopia. Debre Tabor is the administrative town of the South Gondar Zone, which is located 103 km away from Bahir Dar and 667 km Northwest of Addis Ababa. The town has one hospital, known as Debre Tabor Comprehensive Specialized Hospital (DTCSH), and three health centers, namely Debre Tabor Health Center, Leul Alemayehu Health Center, and Atse Seife Areid Health Center, and four health posts. These public health institutions are currently providing various health services, including antenatal care (ANC) services, for the residents of Debre Tabor and the people around the town.

### Population

All pregnant women who had attended the MCH clinic of Debre Tabor public health institutions for ANC service were considered as source population. All pregnant women who came for ANC visits in the selected health institutions during the study period were taken as the study population.

### Eligibility criteria

All volunteer pregnant women (aged 18 years or above) who came for ANC visits in Debre Tabor public health institutions during the data collection period were eligible to participate in the study. However, pregnant women who had serious medical illnesses (severe hypertension, diabetes mellitus, cardiac illness, kidney diseases, and or liver diseases), severe pregnancy-related conditions (antepartum hemorrhage, pre-eclampsia, hyperemesis gravidarum, premature rupture of membrane), and/or serious psychiatric illnesses (psychotic disorder, major depressive disorder, or anxiety disorder) were excluded from the study. Besides, pregnant women aged <18 years were excluded from the study since their educational level is lower and their knowledge, attitude, and decision-making ability about COVID-19 vaccination are most likely different from that of older women.

### Study variables

While COVID-19 vaccine uptake was taken as dependent variables, socio-demographic factors (age, marital status, religion, ethnicity, education, occupation, and residence), obstetric and medical-related variables (gravidity, parity, number of ANC visits, chronic illness, history of contact with COVID-19 cases, history of COVID-19, family history of COVID-19, testing for COVID-19), COVID-19 vaccine knowledge and attitude were considered as independent variables.

### Sample size determination and sampling procedures

The sample size was calculated using the formula shown below by considering Z_α/2_ at a 95% confidence level = 1.96 and margin of error (d)=0.05, with the assumption of 50% COVID-19 vaccine uptake (P) due to lack of prior related study done in Ethiopia.


$$\begin{array}{l} n=\frac{{\left(Z_{1-\alpha /2}\right)}^{2}\;P\left(1-P\right)}{d^{2}}\;*\;Deff \end{array}$$

Design effect (Deff) was calculated using a formula; Deff =1+(m−1) ICC; where m is the average cluster size and ICC is the **i**ntra-cluster correlation coefficient. While m was calculated to be 18, ICC was determined to be 0.03 through a pilot study [ICC= the ratio of the variability between cluster (S^2^b) to the sum of variability between cluster (S^2^b) and variability within-cluster (S^2^w)], making Deff=1.5. Therefore, after multiplying with Deff of 1.5 and adding a 10% non-response rate, the final sample size (n) became 634. A multistage sampling technique was employed to select study participants. Out of all public health institutions in Debre Tabor Town, DTCSH, Debre Tabor Health Center, Leul Alemayehu Health Center, and Atse Seife Areid Health Center were selected using a lottery method. Then the total sample size was proportionally allocated for each health institution. Accordingly, 381, 96, 82, and 75 participants were taken from DTCSH, Debre Tabor Health Center, Leul Alemayehu Health Center, and Atse Seife Areid Health Center, respectively. Then consecutive sampling technique was employed to select the study participants from each health institution during the data collection period.

### Data collection instruments and procedures

Data were collected using a structured questionnaire prepared by adopting different related literature (5, 39). The questionnaire had five parts: Part I: socio-demographic characteristics, Part II: Obstetric and medical-related characteristics; Part III: Knowledge about COVID-19 vaccine; Part IV: attitude toward COVID-19 vaccine, and Part V: COVID-19 vaccination history. Data collection was done (under the supervision of two supervisors) by four BSc nurses who were assigned to their routine work at the MCH clinic of each health institution during the study period.

### Operational definition

#### COVID-19 vaccine uptake

In this study, vaccine uptake was defined as the number of participants who had taken at least one dose of a COVID-19 vaccine at the time of the data collection period. It was measured by the closed-ended question as “Have you ever been vaccinated with any of COVID-19 vaccines at least once recently?” and the response was “Yes” or “No”. Those study participants who have taken the vaccine at least once (receipts of ≥1 COVID-19 vaccine dose) responded as “Yes” and the vaccination status was categorized as ‘vaccinated', whereas, those who were not vaccinated replied as “No” to the question and their vaccination status was labeled as ‘unvaccinated' (40).

#### Knowledge about the COVID-19 vaccine

An eight-point questions survey module was employed to assess the knowledge of respondents regarding the COVID-19 vaccine. Respondents who answered “yes” for knowledge assessing questions were given a score of 1, while those who responded “no or uncertain” for these questions were given 0. The overall knowledge score was categorized into good knowledge; if participants score the median value or above of the knowledge assessing items, and labeled as having poor knowledge; if participants scored below the midpoint of the scale (5).

#### Attitude toward COVID-19 vaccine

A total of ten questions were used to assess the attitude of participants toward COVID-19 vaccines. Respondents who answered “agree” for attitude assessing questions were scored 1 and respondents who answered “disagree or neutral” for attitude assessing questions were given a score of 0. Based on the median score of their responses, participants were labeled as having a positive attitude and a negative attitude. Respondents who had scored equal to the median score or above on the attitude assessment questions of the COVID-19 vaccine were considered as having a positive attitude, whereas those who scored less than the median value were classified as having a negative attitude (5).

### Data processing and analysis

Data were collected first and then checked for completeness and internal consistency. Then data entry was done using Epi Info (version 7.2.4.0) and all statistical analysis was done using Stata version 16.0 software. Clopper-Pearson's exact method was used to calculate the 95% binomial confidence interval (CI) of the overall proportion of vaccinated pregnant women. Simple and multiple logistic regression models were used to examine the factors associated with COVID-19 vaccine uptake. Predictor variables with *p* ≤ 0.25 in simple logistic regression were considered to be candidates in the multiple regression models. Hosmer-Lemeshow test was used to determine the goodness of fit of the logistic regression model. Multiple logistic regression was used to analyze the association between the outcome variable and predictor variables. In multiple logistic regression, the backward variable selection method was used in the analysis. A two-sided *p* < 0.05 and Adjusted Odds Ratio (AOR) at 95% CI were used to consider statistically significant predictors of the outcome variable.

### Data quality assurance

The questionnaire was prepared in English and translated to the local Amharic, and then retranslated back to the English version to ensure consistency. Questionnaires were reviewed by a panel of experts for construct and content validity. Then appropriate modifications, such as correction of wording, logical sequence, inconsistencies, and errors in the skip pattern before the commencement of the actual data collection were made. In addition, to ascertain the understanding, validity, and reliability of the questionnaire and to examine practical issues in selecting participants, a pilot study was conducted before the actual data collection period among 25 pregnant women attending Woreta Health Center. The internal consistency of questionnaires in our study setting was assessed using Cronbach's α. The Cronbach's α for COVID-19 vaccine knowledge, attitude, and uptake questions were 0.81, 0.79, and 0.83 respectively, indicating that the questionnaires have scientifically acceptable internal consistency to measure the knowledge, attitude, and uptake of COVID-19 vaccines among study participants. Moreover, extensive training that lasted for 1 day was given to the data collectors and supervisors on the objectives of the study, the content of the measuring tool, confidentiality, and informed consent. Besides, the data was collected under supervision to ensure the quality of data. The questionnaires were reviewed and checked daily for the completeness of the collected data.

## Results

### Socio-demographic characteristics

A total of 634 eligible participants were included in this study, making the response rate 100%. The mean (±SD) age of participants was 32.3 ± 4.14 years and ranged between 18 and 50 years. The majority of respondents were Orthodox in religion, 613 (96.7%), married, 618(97.5%), and Amhara in ethnicity, 625(98.6%). About 251(39.6%) of respondents had attained primary education and a sizable portion of them were housewives, 281(44.3%), and urban dwellers, 510 (80.4%) (Table 1).

**Table 1:** Socio-demographic characteristics of pregnant women attending antenatal care in Debre Tabor public health institutions, Northwest Ethiopia, 2022.

**Variable**	**Category**	**Frequency (*****n*** = **634)**	**Percent (%)**
Age (years)	<25	70	11.0
	25–34	236	37.2
	35–44	271	42.7
	≥45	58	9.1
Religion	Orthodox	613	96.7
	Muslim	12	1.9
	Protestant	9	1.4
Marital status	Married	618	97.5
	Single	6	0.9
	Divorced	9	1.4
	Widowed	1	0.2
Educational status	No formal education	147	23.2
	Primary education	251	39.6
	Secondary education	203	32.0
	College/University	33	5.2
Occupation	Housewife	281	44.3
	Merchant	222	35.0
	Government employee	104	16.4
	Private employee	18	2.8
	Student	6	1.0
	Daily laborer	3	0.5
Ethnicity	Amhara	625	98.6
	Tigray	4	0.6
	Oromo	3	0.5
	Other	2	0.3
Residence	Urban	510	80.4
	Rural	124	19.6


### Obstetric and medical-related characteristics

The majority 557(87.9%) of women were multigravida having two or more pregnancy history including the current one. Only 79(12.4%) of participants never had a live birth (nulliparous), but the rest majority 555(87.6%) had a prior history of at least one live birth (primiparous or multiparous). The current pregnancy of nearly 623 (98.3%) study participants was planned. Almost half 313(49.4%) of them were in the third trimester of the current pregnancy. The greatest proportion 24(58.1%) of pregnant women had fewer than four ANC visits at the time of the interview.

A considerable proportion 585 (92.3%) of participants had no known history of contact with confirmed COVID-19 cases. Around 42 (6.6%) respondents had a prior history of COVID-19 infection, while 47 (7.4%) of them had a family history of COVID-19 infection. About 41 (6.5%) of all participants were tested for COVID-19, and 68.3% of those tested were confirmed positive. About 12(1.9%) of participants had a history of chronic medical illness, with hypertension (41.7%) followed by diabetes mellitus (33.3%) reported to be the most prevalent chronic diseases (Table 2).

**Table 2:** Obstetric and medical related characteristics of pregnant women attending antenatal care in Debre Tabor public health institutions, Northwest Ethiopia, 2022.

**Variables**	**Category**	**Frequency (*n* = 634)**	**Percent (%)**
Gravidity	Primigravida	77	12.1
	Multigravida	557	87.9
Parity	Nulliparous	79	12.4
	Primiparous	119	18.8
	Multiparous	436	68.8
Planned (current) pregnancy	Yes	623	98.3
	No	11	1.7
Trimester of the current pregnancy	First trimester	75	11.8
	Second trimester	246	38.8
	Third trimester	313	49.4
Number of ANC visits	<4	368	58.1
	≥4	266	41.9
History of contact with confirmed COVID-19 cases	Yes	49	7.7
	No	585	92.3
Prior history of COVID-19 infection	Yes	42	6.6
	No	592	90.4
Family history of COVID-19 infection	Yes	47	7.4
	No	587	92.6
Tested for COVID-19 infection	Yes	41	6.5
	No	593	93.5
COVID-19 test result (*n* = 41)	Positive	28	68.3
	Negative	13	31.7
Chronic medical illness	Yes	12	1.9
	No	622	98.1
Types of chronic illness (*n* = 12)	Hypertension	5	41.7
	Diabetes mellitus	4	33.3
	Heart disease	2	16.7
	Kidney disease	1	8.3


### Knowledge of respondents about COVID-19 vaccine

**Figure 1:**
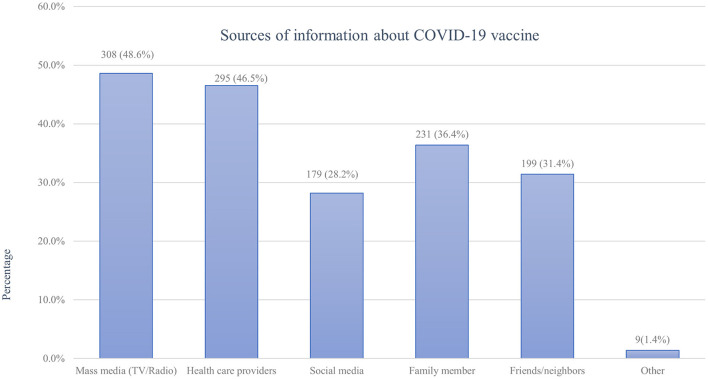
Sources of information about the COVID-19 vaccine among pregnant women attending antenatal care in Debre Tabor public health institutions, Northwest Ethiopia, 2022.

All study participants (100%) claimed that they heard about COVD 19 vaccine from different information sources. Their main source of information regarding COVID-19 vaccines was mass media such as TV and radio followed by health care providers and family members, accounting for 308(48.6%), 295 (46.5%), and 231 (36.4%) respectively (Figure 1).

The overall knowledge of the respondents about the COVID-19 vaccine was assessed using an eight knowledge assessing questions. Accordingly, more than half 356(56.2%) of respondents were evaluated to score the median value or above and were labeled as having good knowledge. But the remaining 278(43.8%) were below the median score and considered to have poor COVID-19 vaccine knowledge. About 47.6% of respondents replied that pregnant women need to get the COVID-19 vaccination. Besides, more than two-thirds (68.1%) of them correctly responded as a vaccine could protect against COVID 19. Participants also answered that COVID-19 vaccines produce long-term immunity (58.4%), reduce disease severity (51.3%), have no health-related risk (49.0%), and carry no risk of harm to the baby (39.4%) (Supplementary Table 1).

### The attitude of respondents toward COVID-19 vaccine

A total of ten questions were used to evaluate the overall attitude of participants toward COVID-19 vaccines. Thus, 264(41.6%) of pregnant women were scored equal to the median or above and categorized as having a positive attitude toward the COVID-19 vaccine, whereas more than half 370(58.4%) of them scored below the median and classified as having a negative attitude. Specifically, the belief of participants COVID-19 vaccine is essential (73.8%) and currently accessible for all population (75.4%) were the highest scoring item. Whereas, vaccination reduces the risk of getting COVID-19 (48.6%), reduces the incidence of COVID-19 (42.8%), protects against COVID-19 complications during pregnancy (41.8%), is safe 234(36.9%), and is effective (43.4%) were the lowest scoring items of the attitude assessing questions (Supplementary Table 2).

### COVID-19 vaccination history

**Figure 2:**
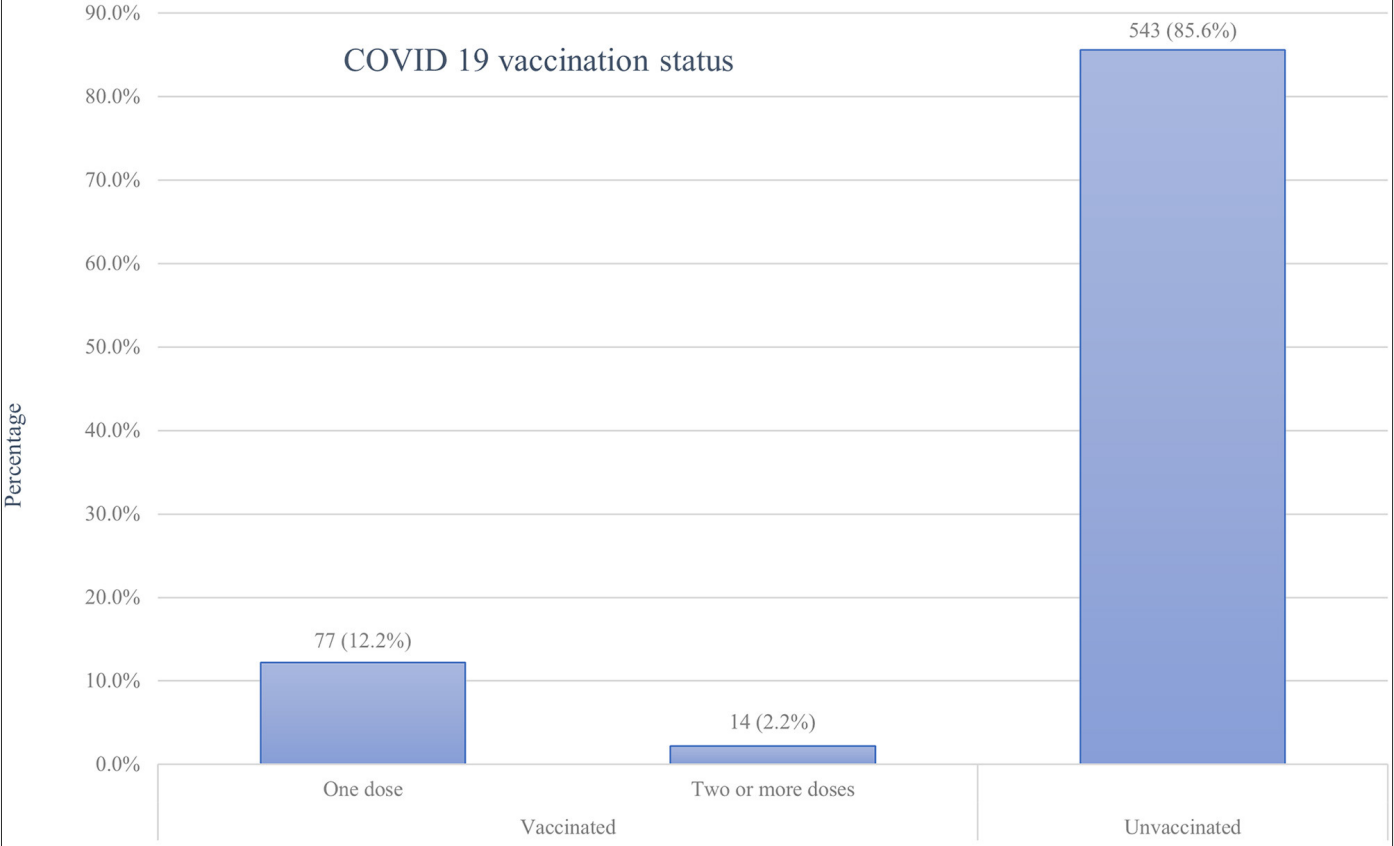
COVID-19 vaccination status of pregnant women attending antenatal care in Debre Tabor public health institutions, Northwest Ethiopia, 2022.

**Figure 3:**
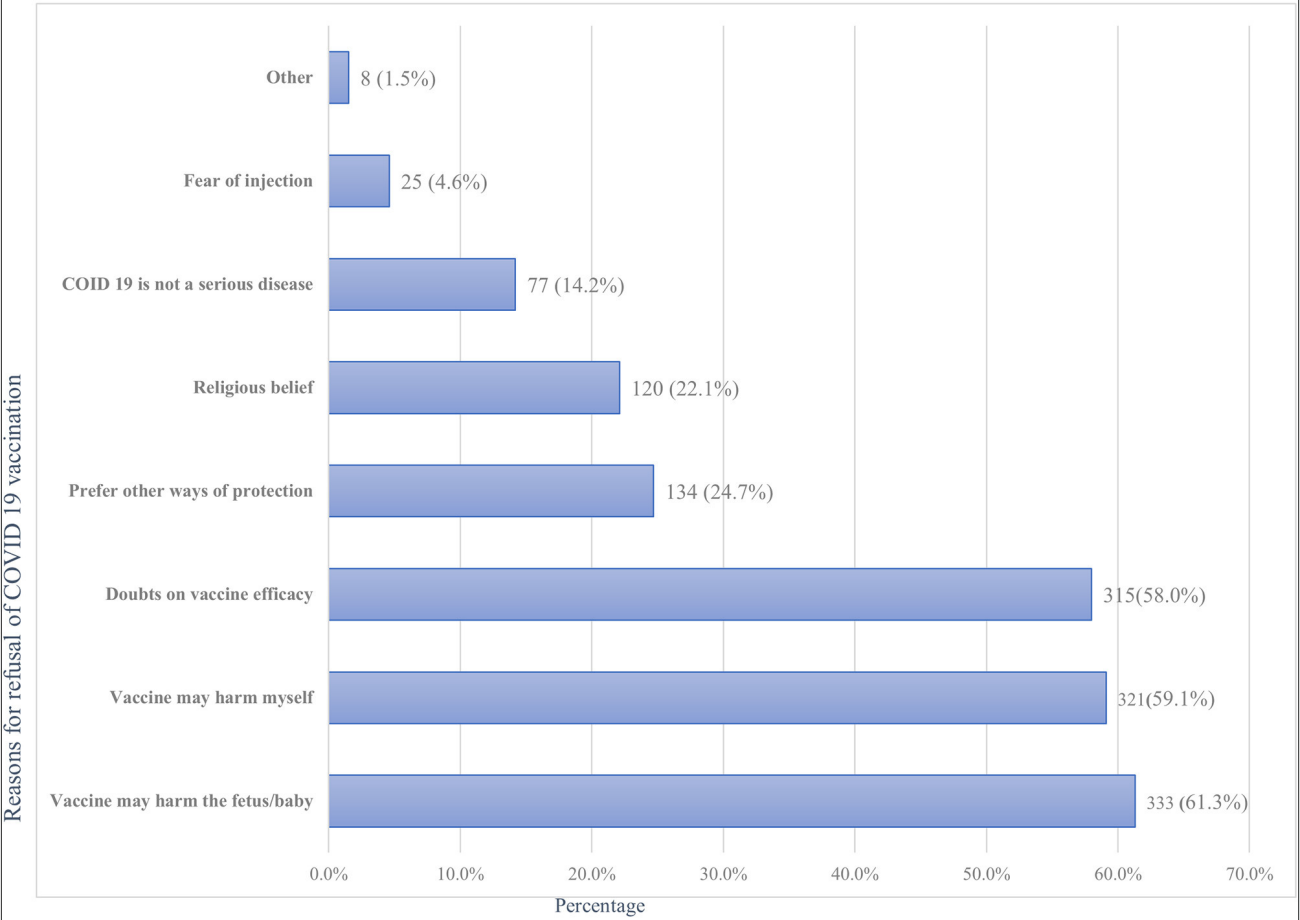
Reasons for refusal of the COVID-19 vaccination among pregnant women attending antenatal care in Debre Tabor public health institutions, Northwest Ethiopia, 2022.

Of all the respondents, about 91 (14.4%; 95%CI: 11.7%-17.3%) of them had taken at least one dose of the COVID-19 vaccine, with only a minority (2.2%) of all samples being fully vaccinated (Figure 2). Of those vaccinated, the majority 59 (64.8%) did not experience any post-vaccination symptoms, while a few 32(35.2%) faced minor side effects. Fever 13(40.6%), fatigue 12(37.5%), and headache 9 (28.1%) were the main symptoms reported by the participants. Symptoms such as joint pain 6 (18.8%), myalgia 6 (18.8%), chills 5 (15.6%), and others 2 (6.3%) were the rare side effects among the respondents. However, the vast majority of participants, 543 (85.6%), were not vaccinated with any of the COVID-19 vaccines for various reasons. Fear of side effects due to the perception that vaccines may harm their baby (61.3%) or themselves (59.1%) and doubts about vaccine efficacy (58.0%) were the most frequent reasons for denying COVID-19 vaccination (Figure 3).

### Factors associated with COVID-19 vaccine uptake

The association between independent variables and COVID-19 vaccine uptake was analyzed using binary logistic regression. A simple logistic regression model was first used and variables with p ≤ 0.25 were proceeded into multiple logistic regression models to adjust covariates. Based on adjusted logistic regression analysis, age, marital status, educational status, vaccine knowledge, and attitude were significantly associated with COVID-19 vaccine uptake (Table 3). The odds of COVID-19 vaccine uptake in pregnant women aged 45 years or above was 1.75 times (AOR: 1.75, 95%CI: 1.01, 2.95) higher than in those who were under 25 years. Compared to unmarried women, married pregnant women had 1.26-fold (AOR: 1.26, 95%CI: 1.12, 2.96) higher odds of being vaccinated. Pregnant women who had completed college or university education were a 57% (AOR: 0.43, 95%CI: 0.12–0.69) lower likelihood of COVID-19 vaccine uptake than those with no formal education. COVID-19 vaccination was 3.52 times (AOR:3.52, 95%CI:1.83–3.87) higher likelihood in participants with good knowledge than those with poor knowledge. Whereas, respondents with a positive attitude toward the COVID-19 vaccine had 4.81-fold (AOR:4.81, 95% CI:1.42–7.33) more likely to receive the COVID-19 vaccine than their counterparts.

**Table 3:** Factors associated with COVID-19 vaccine uptake among pregnant women attending antenatal care in Debre Tabor public health institutions, Northwest Ethiopia, 2022.

**Variable**		**Vaccine uptake, n(%)**		**COR (95%CI)**	**AOR (95%CI)**
		**Yes (91)**	**No (543)**		
Age (in years)	<25	8 (8.8%)	62 (11.4%)	1	1
	25–34	30 (32.9%)	206 (37.9%)	0.89 (0.73–1.79)	0.75 (0.69–2.67)
	35–44	35(38.5%)	236(43.5%)	1.44 (1.06–3.21)	1.12 (0.94–4.21)
	≥45	18(19.8%)	40 (7.4%)	2.34 (1.21–4.58)*	1.75 (1.01–2.95)*
Marital status	Married	89(97.8%)	529(97.4%)	1.27 (0.89–1.89)	1.26 (1.12–2.96)*
	Unmarried^a^	2 (2.2%)	14 (2.6%)	1	1
Educational status	No formal education	29 (31.8%)	118 (21.7%)	1	1
	Primary education	39 (42.9%)	212(39.0%)	0.92 (0.42–1.97)	0.98 (0.59–2.33)
	Secondary education	20 (22.0%)	183 (33.7%)	0.83 (0.49–0.99)*	0.77 (0.54–1.78)
	College/University	3(3.3%)	30 (5.5%)	0.32 (0.23–0.85)*	0.43 (0.12–0.69)*
Occupation	Housewife	38 (41.8%)	243(44.8%)	1	1
	Other^b^	53 (58.2)	300 (55.2%)	1.44 (0.86–2.13)	1.82 (0.97–2.39)
Residence	Urban	66 (72.5%)	444 (81.8%)	0.93 (0.32–1.26)	0.82 (0.29–1.98)
	Rural	25 (27.5%)	99 (18.2%)	1	1
Gravidity	Primigravida	5(5.5%)	72 (13.3%)	1	1
	Multigravida	86(94.5%)	471(86.7%)	1.54 (0.35–4.32)	0.57 (0.45–2.37)
Parity	Null parous	4(4.4%)	75(13.8%)	1	1
	Primiparous	18(19.8%)	101(18.6%)	0.88 (0.75–2.58)	0.56 (0.26–2.18)
	Multiparous	69 (75.8%)	367(67.6%)	1.33 (0.67–2.71)	1.55 (0.89–2.87)
Number of ANC visit	<4	50 (54.9%)	318(58.6%)	1	1
	≥4	41 (45.1%)	225(41.4%)	0.66 (0.54–3.17)	0.85 (0.77–1.73)
Current pregnancy	Planned	88(96.7%)	535(98.5%)	1.44 (0.89–2.38)	1.35 (0.22–2.43)
	Unplanned	3 (3.3%)	8(1.5%)	1	1
History of contact with COVID-19 cases	Yes	8(8.8%)	41 (7.6%)	1.16 (1.00–1.35)	1.2 (0.11–2.15)
	No	83(15.3%)	502(92.4%)	1	1
History of COVID-19	Yes	7(7.7%)	35(6.4%)	1.25 (0.92–2.74)	1.6 (0.71–2.48)
	No	84 (92.3%)	508(93.6%)	1	1
Family history of COVID-19 infection	Yes	5(5.5%)	42 (7.7%)	0.91 (0.67–1.46)	1.44 (0.98–3.21)
	No	86(94.5%)	501 (92.3%)	1	1
Tested for COVID-19	Yes	5(5.5%)	36 (6.6%)	0.71 (0.63–4.84)	0.33 (0.21–1.39
	No	86(94.5%)	507 (93.4%)	1	1
Chronic diseases	Yes	2(2.2%)	10(1.8%)	0.95 (0.89–2.91)	1.03 (0.64–3.91)
	No	89(97.8%)	533(98.2%)	1	1
Knowledge	Good knowledge	62(68.1%)	294(54.1%)	1.57 (1.18–2.91)*	3.52 (1.83–3.87)**
	Poor knowledge	29(31.9%)	249(45.9%)	1	1
Attitude	Positive attitude	57 (62.6%)	207(38.1%)	3.76 (1.51–3.99)**	4.81 (1.42–7.33)**
	Negative attitude	34 (37.4%)	336(62.9%)	1	1

Significant at *p < 0.05, **p < 0.001; 1 = reference category.

*^a^single, divorced, and widowed*;

*^b^government employee, private employee, merchant, daily laborer & student*.

*COR, Crude odds ratio; AOR, Adjusted odds ratio; CI, Confidence interval*.

## Discussion

According to the current study, only 14.4% of pregnant women were vaccinated for COVID-19 at least once. This figure is significantly lower than the findings from similar studies conducted in other countries, such as Saudi Arabia (57.1%) (10), Scotland (32.3%) (31), the US (40%) (29), and England (53.7%) (38). This disparity could be attributable to the increased COVID-19 morbidity and mortality rates, as well as improved socioeconomic status, which could promote vaccine uptake among pregnant women in these countries. The late delivery of the vaccine in Ethiopia, as well as a considerably low level of knowledge and attitude toward the COVID-19 vaccination in our study setting, could also be the contributing factors to lowered vaccine uptake.

The vaccine uptake among pregnant women in this study was also lower than other parts of the population in Ethiopia (40, 41). According to a web-based study done among Ethiopian health professionals, 62.1% of them had received the COVID-19 vaccine (40). A previous cross-sectional study was done in Eastern Ethiopia among people aged 50 and above indicates that 39.4% of the participants had taken the vaccine at least once (41). In this context, the lower rate of vaccination among pregnant women than in these population groups could be explained by the late commencement of COVID-19 vaccination in the general population. COVID-19 vaccination for health professionals and the elderly (>50 years) began on 13 March 2021, which was earlier than the general public (including expecting mothers) launched on 16 November 2021. This was due to the government's prioritization to vaccinate high-risk groups over others as a result of the vaccine shortage. COVID-19 vaccine uptake among pregnant women in this study was also significantly lower than receipts of the vaccine among Ethiopia's general population. Preliminary national data from Ethiopia showed that approximately 25.1% of the population has received at least one dose of the COVID-19 vaccine (42). This reveals that pregnant women are more vaccine-resistant than other parts of the population. This is supported by a study done in the UK, showing that pregnant women were more likely to be vaccine-resistant than non-pregnant individuals (43). Consistently, other reports documented that pregnant women were less likely to complete the COVID-19 vaccine series than non-pregnant women, and their vaccination rates remained low (20, 29). This might be due to higher vaccine hesitancy among pregnant women compared to other parts of the population due to vaccine safety concerns, misconceptions, and fear of harm to the fetus (5, 10).

In the current study, the most common reasons for refusing COVID-19 vaccination were vaccine safety concerns due to the fear of harmful side effects for the fetus or the mother themselves, which is supported by many other studies (37, 44–46). Our study reported that a considerable proportion of respondents were highly concerned about vaccine safety and hence declined COVID vaccination. Similarly, several previous studies have found that a sizable proportion of participants were concerned about the safety of vaccines during pregnancy (36, 37, 47). Taken together, pregnant women are still highly concerned about vaccine safety and refuse to take the vaccine despite the fact that major guidelines reported the seriousness of COVID-19 and the safety of the COVID vaccine during pregnancy (14, 15, 20). Prior studies indicated that the majority of COVID-19 vaccinated pregnant people had no serious post-vaccination symptoms but mild to moderate side effects may sometimes occur in pregnant women similar to nonpregnant women (20, 22). In agreement, our study results demonstrated that the majority of participants did not experience any post-vaccination symptoms. However, a few most commonly reported minor symptoms were fever, headache, fatigue, joint pain, myalgia, and chills, with no serious post-vaccination pregnancy-related adverse effects. This is in line with Kadali et al., which reported that sore arm, fatigue, headache, chills, myalgia, nausea, fever, and sweating are the most frequent side effects reported by both pregnant and non-pregnant women at the same rates (48).

We have also found that age, marital status, educational status, knowledge, and attitude were significantly associated with COVID-19 vaccine uptake. It was shown that COVID-19 vaccine uptake was significantly increased in older pregnant women than in younger women. This agrees with prior studies in the UK (49), Scotland (31), Ethiopia (39, 40), Saudi Arabia (10), Jordan (34), the US (50), and Bangladesh (51). This is possibly due to the fact that there is an increment in the understanding of COVID-19 risk among the elderly. But the contradicting result was reported from another study done in Bangladesh, where young people took the vaccine more than old people (52). More interestingly, married pregnant women were found to have higher odds of being vaccinated than unmarried ones. This is potentially due to marriage may increase women's empowerment and decision-making ability to receive the vaccine. Married women might be more financially stable and are more likely to get vaccinated than unmarried women. Compared to unmarried women, this subset of the population may have a greater level of husbands' support, which increases the likelihood of receiving vaccination. This result, however, disagrees with other studies done in Ethiopia (39, 41).

Furthermore, our study showed that COVID-19 vaccine uptake was lower among participants who had attained college or university education than those with no formal education. This is supported by prior studies, revealing that people who had no education were more likely to be vaccinated than people who had attended above secondary school (41, 53). This might be due to uneducated people being more likely to receive the vaccine without considering the possible adverse effects of the vaccine, while the educated people could have more awareness about the vaccine preparation time, safety, and side effects and may hesitate to take the vaccine. But our finding contradicts other studies that show educated people are more likely to get vaccinated than uneducated people (54–56). But some other studies did not observe any significant association between educational status and COVID-19 vaccination (5, 40).

In addition, this study showed that good knowledge of the COVID-19 vaccine was a significant predictor of the COVID-19 vaccination. This is congruent with several prior studies, which indicate a significant association between vaccine knowledge, intention, and uptake (37, 57–60). This could be explained by the theory of the knowledge-attitude-behavior paradigm, which assumes that knowledge of individuals' health is a key factor to engage in a particular health-related behavior (61). Pregnant women with good COVID-19 vaccine knowledge, in particular, can better understand its potential benefits, resulting in favorable vaccine beliefs and increased vaccine trust and uptake (62, 63). But those with poor knowledge are more likely to associate the vaccines with side effects and believe in misinformation about vaccine safety, potentially increasing the perceived risk of vaccine side effects and refusing of COVID-19 vaccination (64–66). This implies that imparting relevant knowledge about the COVID-19 vaccine matters for vaccination (67). We also found that participants with a positive attitude toward the COVID-19 vaccine had a higher likelihood of taking the COVID-19 vaccine than those with a negative attitude, which is supported by other prior studies (5, 37, 60). This could be explained by the existing theory that individuals' attitude influences their health behavior (61, 68, 69). Thus, attitudes on vaccine adverse effects, safety, and efficacy might influence the willingness and practice of vaccination during pregnancy (5, 70, 71). Expectant women with a positive attitude may trust the vaccines and follow the instructions provided by various guidelines, making them more likely to receive the vaccine.

Despite our best efforts, there are some limitations to our study. Since the study employed a cross-sectional study design, it might not show the temporal relationship between cause and effect. The study may have limited representativeness as it was conducted in institutions.

## Conclusion

The COVID-19 vaccine uptake among pregnant women in this study was very low. The main reasons for low COVID-19 vaccination rates were safety concerns due to the fear that the COVID-19 vaccine may have harmful side effects to the fetus or the mothers themselves. Older age, no formal education, good knowledge, and positive attitude of pregnant women were independent predictors of COVID-19 vaccine uptake. Thus, extensive awareness creation campaigns should be undertaken using different media of communication by providing special consideration for pregnant women to address misunderstandings on adverse effects, vaccine safety, and hesitancy. Besides, health care providers must take the opportunity to routinely assess pregnant women's immunization status and to have a discussion about the benefits of COVID-19 vaccines during each ANC visit. This will enhance the knowledge about and the attitude toward COVID-19 vaccine and draw more attention to promote public trust in the COVID-19 vaccine, thereby increasing the willingness to accept vaccination. In addition, further large-scale clinical studies need to be conducted on the safety and potential side effects associated with vaccination during pregnancy.

## Data Availability Statement

The original contributions presented in the study are included in the article/Supplementary Material, further inquiries can be directed to the corresponding author.

## Ethics Statement

The studies involving human participants were reviewed and approved by Ethical Review Committee of Debre Tabor University. The patients/participants provided their written informed consent to participate in this study.

## Author contributions

All authors made a significant contribution to the work reported, whether that is in the conception, study design, execution, acquisition of data, analysis and interpretation, or in all these areas, took part in drafting, revising, or critically reviewing the article, gave final approval of the version to be published, have agreed on the journal to which the article has been submitted, and agree to be accountable for all aspects of the work.

## Conflict of interest

The authors declare that the research was conducted in the absence of any commercial or financial relationships that could be construed as a potential conflict of interest.

## Publisher's note

All claims expressed in this article are solely those of the authors and do not necessarily represent those of their affiliated organizations, or those of the publisher, the editors and the reviewers. Any product that may be evaluated in this article, or claim that may be made by its manufacturer, is not guaranteed or endorsed by the publisher.

## Abbreviations

ANC: Antenatal care
AOR: Adjusted Odds Ratio
CI: Confidence Interval
COVID 19: Coronavirus Disease 19
DTCSH: Debre Tabor Comprehensive Specialized Hospital
ICU: intensive care unit
MCH: Maternal and Child Health
SARS-CoV-2
Severe Acute Respiratory Syndrome Coronavirus-2
SD: Standard Deviation
WHO: World Health Organization.
