# Supplementary material for: COVID-19 vaccine uptake and associated factors among pregnant women attending antenatal care in Debre Tabor public health institutions: A cross-sectional study
Source: Front Public Health. 2022 Jul 19;10:919494. doi: 10.3389/fpubh.2022.919494 (PMC9343782; doi:10.3389/fpubh.2022.919494)
Supplement: Supplementary file 1 [file Data_Sheet_1.docx]

**Supplementary files**

COVID 19 vaccine uptake and associated factors among pregnant women attending antenatal care in Debre Tabor public health institutions: A cross sectional study

Endeshaw Chekol Abebe^1^|Gebrehiwot Ayalew Tiruneh ^2^|Getachew Asmare Adela ^3^|Teklie Mengie Ayele^4^|Zelalem Tilahun Muche^1^|Awgichew Behaile T/Mariam^1^|Anemut Tilahun Mulu^1^|Edgeit Abebe Zewde^1^|Fitalew Tadele Admasu^1^|Nega Dagnaw Baye^1^|Tadesse Asmamaw Dejenie^5^

**Supplementary Table 1:** Knowledge about the COVID 19 vaccine among pregnant women attending antenatal care in Debre Tabor public health institutions, Northwest Ethiopia, 2022

| Items | Responses, n (%) | | |
| --- | --- | --- | --- |
|  | Yes | No | Uncertain |
| Heard about COVID 19 vaccine | 634 (100.0% | 0(0.0%) | 0(0.0%) |
| Pregnant women need to get COVID 19 vaccination | 302(47.6%) | 219 (34.6%) | 113(17.8%) |
| Vaccines protect against COVID 19 | 432 (68.1%) | 66 (10.4%) | 136 (21.5%) |
| COVID 19 vaccines produce long term-immunity | 370(58.4%) | 86(13.6%) | 178(28.0%) |
| COVID 19 vaccines can reduce disease severity | 325(51.3%) | 79(12.5%) | 230(36.2%) |
| COVID 19 vaccine have no health-related risk | 311(49.0%) | 121(19.1%) | 202(31.9%) |
| COVID-19 vaccine carries no harm to your baby | 250(39.4%) | 172(27.1%) | 212(33.5%) |
| COVID 19 vaccination is launched in Ethiopia | 611(96.4%) | 7(1.1%) | 16(2.5%) |

**Supplementary Table 2:** Attitude towards the COVID 19 vaccine among pregnant women attending antenatal care in Debre Tabor public health institutions, Northwest Ethiopia, 2022

| Items | Responses, n (%) | | |
| --- | --- | --- | --- |
|  | Agree | Neutral | Disagree |
| Do you believe COVID-19 vaccine is essential? | 468(73.8%) | 122(19.3%) | 44(6.9%) |
| Do you think vaccine reduces the chance of getting COVID-19? | 308(48.6%) | 192 (30.3%) | 134(21.1%) |
| Do you believe vaccine lower the risks of COVID-19 complication? | 333(52.5%) | 188(29.7%) | 113(17.8%) |
| Do you think the information given by official media on COVID 19 vaccine is reliable? | 349(55.0%) | 216(34.1%) | 69(10.9%) |
| Do you think the current COVID-19 vaccine is safe? | 234(36.9%) | 287(45.3%) | 113(17.8%) |
| Do you believe the current COVID-19 vaccine is effective? | 275(43.4%) | 278(43.8%) | 81(12.8%) |
| Do you believe COVID-19 vaccination can reduce the spread of the virus in the community? | 304(48.0%) | 233(36.7%) | 97(15.3%) |
| Do you believe COVID-19 vaccines are currently accessible for all population? | 478(75.4%) | 98(15.5%) | 58(9.1%) |
| Is it possible to reduce the incidence of COVID-19 with vaccination? | 271(42.8%) | 212(33.4%) | 151(23.8%) |
| Do you believe the current vaccine protect against COVID 19 complications during pregnancy | 265(41.8%) | 219(34.5%) | 131(20.7%) |
